# The Relation and Hereditary Tendency of Inebriety and Epilepsy

**Published:** 1877-04

**Authors:** Edward C. Mann

**Affiliations:** Late Medical Superintendent of State Emigrant Insane Asylum, Ward’s Island, New York


The Relation and Hereditary Tendency of Inebriety and Epilepsy.—*By Edward C. Mann, M.D., Late Medical Superintendent of State Emigrant Insane Asylum, Ward's Island, New York.*—Very little attention has as yet been devoted to the relation and hereditary tendency existing between inebriety and epilepsy, although a very close relation undoubtedly exists between them. Careful examination reveals a large number of persons affected with epilepsy whose parents or ancestors have been addicted to intemperance. There is a very close analogy existing between the paroxysms of a dipsomaniac, where there is often a prodromic stage of nervous disturbance which may incapacitate the patient for mental labor, and the convulsions of an epileptic, whose paroxysms of intense nervous excitement are generally preceded by the "aura epileptica;" the difference being, that in the former case the paroxysm lasts for weeks, perhaps, while in the case of the epileptic it lasts but a few moments. As we often see the two diseases existing in the same person, it becomes impossible not to infer a similarity of origin. We have in both instances accumulated and pent-up nervous force or irritation, which finally expends itself, in the one case, in the unrestrained indulgence—in the irresistible impulse—to indulge in alcoholic stimulants, and in the other, in the convulsive movements of epilepsy. There would seem, beyond all doubt, to be a correlation of force which results in the mutual convertibility of these two diseases. It is not an unusual case to find in the various members of different generations of the same family, different phases of the neuroses, such as insanity, epilepsy, phthisis, chorea, or inebriety, showing, beyond all doubt, correlation of morbific force in hereditary diseases. I believe most firmly that the morbid condition of nerve element, or morbific force induced by inebriety, is indelibly impressed upon, or is transmitted to, the ovum at the time of conception, and that this morbific force lies dormant in the system until developed by an adequate exciting cause, and that the hereditary neurosis thus often skips a generation, leaving no appreciable manifestation of its existence in the intermediate generation. When this morbific force *does* manifest itself, next to the transmission of the pre-disposition to inebriety, comes, unquestionably, epilepsy. From my experience, the children or grandchildren, while infants, are generally affected with convulsions, which may prove fatal, but more often tend to assume an epileptiform type as the child advances in years. I have repeatedly noticed in patients who did not have complete epileptic seizures, *epileptic vertigo*, which passed off almost instantly, but which for the time evidently abolished consciousness, partially, if not entirely. The brain of such children is often morbidly active, and too high pressure in education, or an unnatural forcing process during the formative period of childhood, often results, especially in girls, during the period of constitutional evolution—a time at which the organism is under physiological conditions that pre-dispose to pathological states—in disturbances, primarily, of the organs of respiration, circulation, and digestion; and secondarily, in the production of hysteria and epilepsy, by overstimulating a brain already morbidly active and pre-disposed to disease upon the application of even comparatively trifling exciting cause. Again, consanguinous marriages may be the connecting link between inebriety and epilepsy. I have known cases in which the intermarriage of blood relations, where there was inebriety that had laid dormant for one or two generations, has resulted in the old hereditary neurosis reappearing in the form of epilepsy in the offspring. It is a curious fact, also, that the sons born as the result of union of cousins in marriage, appear to have a strong tendency towards inebriety. The only explanation which I can offer of such cases is, that it is probably the development of the latent morbific force residing in the constitution of the parents, who have a common ancestor, which has been lying dormant for one or two generations, and which is developed in the offspring as the result of the consanguineous marriage. I think that such latent morbific force and hereditary disease is far more common than we generally suppose, and that in many cases, both insanity and inebriety are only the expression of latent disease, elicited by external, accidental causes, rather than as the result of moral or physical causes, to which they are attributed both by the laity and by the profession; the epileptic convulsions occurring as the result of inebriety, depend mainly, I think, upon a two-fold cause, which operates in the production of a morbid irritability of the medulla oblongata. Whether this be a transitory or a constant state, I think is an open question. I am inclined to think that it is a *constant* state, which may, however, for the production of the epileptic paroxysm, require the additional stimulus of transitory cerebral irritation to be transmitted to the medulla oblongata, and perhaps the sympathetic. The two-fold cause which has appeared to me to operate in the production of what I take leave to term *alcoholic epilepsy*, consists, first, in the hyperæmia of the brain, which causes symptoms of irritation, due to increased excitability of the nerve filaments and ganglion cells of the brain, which, by transmitting to the medulla oblongata a morbid irritability, results in epileptiform convulsions; second, a state of cerebral anæmia, which also induces a morbid irritability of the medulla by causing arterial anæmia.

Finally, I believe, that as a result of inebriety, we may have epileptic convulsions occurring, merely from the state of nervous irritation produced in the medulla and at the base of the brain, which excites the motor nerves, from the poisonous and improper character of the blood plasma, produced by the presence in it of alcohol. This is probably often the case, without either very marked anæmia or hyperæmia of the brain. This appears reasonable when we reflect upon the fact that the amount of blood going to the brain constitutes about one-fifth of the whole bulk of the blood. Consequently any poisonous or injurious change in the condition of the usual supply of blood must be very apparent in the encephalic condition, and produces cerebral irritation incompatible with the performance of the healthy function. There is in epilepsy, and more especially in epileptic insanity, a period which sometimes precedes, and sometimes follows the epileptic paroxysm, in which often occurs an abrupt and complete change from a sober, honest, and industrious, to a dissipated, negligent, and lazy man. These attacks often occur periodically in the course of epilepsy, associated with more or less maniacal excitement. During these periods of moral alienation there has often occurred in patients under my charge, an iresistable desire to indulge in alcoholic stimulants to excess, so that for the time, the patient, if not restrained, would pursue a course of inebriety, his reason seeming powerless to control the temporary dipsomania. There is still another class of cases in which the moral insanity takes the place of the epilectic paroxysm, and these cases are also very prone, according to my experience, to be addicted to inebriety if the opportunity of gratifying their morbid impulse occurs. In my observations, and in my study of insanity, I am continually called upon to witness its connection with disease, and more particularly with the hereditary disease, and I have come to believe as I have endeavored to show, that inebriety and epilepsy are mutually convertible diseases. They both depend upon the morbific force, before alluded to, which may remain latent in the nervous system for a long time between the intervals of its manifestation, and although there is a certain dissimilarity in the symptoms between the two diseases, it does not at all follow that they are owing to different causes. The fact of the hereditary disease appearing in different forms in members of the same family and passing from one form to another, leads us to positively infer a correlation of morbific force which leads to this mutual convertibility, which includes not only inebriety and epilepsy, but also, I think, phthisis, skin disease, insanity, scrofula, and perhaps, rheumatism, and gout. In my Asylum practice, I have had ample proof of this, in the existence of the different phases of hereditary disease, and their alternations, both in the same individual and in various members of different generations of the same family. With regard to the treatment of hereditary disease, which I do not propose to take up in this paper, the chief indications point to hygiene and to wise marriages. As we have seen that the symptoms of constitutional diseases are manifested soon after birth in convulsions, or some affection of the nervous system, we must turn our attention to the nourishment and education of the child, and endeavor to exclude everything prejudicial to its future mental and bodily health. By such attention to hygienic rules are we, as physicians, to endeavor to secure an exemption in the rising generation from these hereditary diseases so far as we may, and it is certainly our duty to aim at the eradication of hereditary disease, which, if it is ever accomplished in the future, as we hope it may be, will complete the round of the possibilities of preventive medicine.

Epilepsy occurring in offspring, as the result of inebriety in the progenitors, is complicated with defects or disorders of the mind in various ways, and the manifestations may, with propriety, I think, be classified as follows: 1st. Epileptic idiots, whose intellectual faculties have never been developed. 2d. Epileptics who are imbecile or demented. 3d. Epileptic maniacs, who, without obvious disorder of the mind, when epileptic fits are coming on, are irritable, morose, malicious, and dangerous, and sometimes commit fearful crimes. In some instances the mental disorder takes the form of a paroxysm of acute mania coming on suddenly. 4th. Epileptics whose intellects are not impaired.

*Pathology.* The pathology of the production of epilepsy in the offspring of intemperate parents is very obscure. The condition of the mother during gestation, if abnormal as in inebriety, cannot fail to interfere with the proper nutrition of the cerebral tissue of the fœtus, and it is in this way, I think, that during embryo life, the brain of the infant often undergoes pathological changes, which induce both deficient moral power and epilepsy. It is certain that any pathological state which destroys the equilibrium of the functions of the organs of the mind, producing depression of some functions and excitement of others, cannot fail to produce in the children of such parents, an ill-balanced and defective state of the nervous system, disposed to take on diseased action. It is probable that there exists in such children, a state of the cerebral vessels which interferes with the uniform and healthy interchange of nutritive plasma passing from the vessels to the brain-cells, and of the fluid-cell contents in a state of degenerate metamorphosis, passing from the cells to the vessels. This state of the cerebal capillaries induces a morbid activity of the cerebral cells, which is in all probability the determining cause of the epileptiform convulsions from which such children suffer. That the functional disturbance resulting in epileptiform convulsions in such children may be due to diverse causes, that is, to anæmia as well as hyperæmia, I have proved to be a fact, as I have found, in two or three instances, the brain and membranes completely bloodless, in children who died in hospital in the midst of convulsions, although I think that the general rule in such cases is more often to find an excessive quantity of blood present.

After numerous dissections of the brains of epileptics both in cases resulting from intemperance, and in cases occurring under ordinary conditions, Foville and Andral agree in their testimony that there is no special lesion attending this malady. Andral insists upon the necessity of distinguishing between those cases in which death occurs in the interval, and those in which the patient dies in a fit, as in the latter class of cases there will be congestion of the cerebral vessels, which is the *effect* and not the *cause* of the fit as some might suppose. I have found that in the cases coming under my charge, in which inebriety in the ancestors could be clearly traced as a predisposing cause, very few, if any of the patients, had been healthy persons previous to the occasion of the disease. That in a great many instances, especially in women, hysteria and other nervous affections had existed previously, and that functional derangements of the nervous system had been of frequent occurrence since infancy. The pathological appearances which have been found by Schroeder Van der Kolk in the medulla oblongata, would seem to show that epileptic convulsions depend generally upon an increased influx of blood to the medulla oblongata; although, as I have previously stated, it is a fact which has been proved by the experiments of Kussmaul and Tenner, in cutting off the arterial blood from the brain, that arterial amæmia is also a cause of epilepsy. Schroeder Van der Kolk found that in the medulla there generally existed a dilatation of the arterioles and capillary vessels, with thickening of their coats. In the cases where arterial anæmia of the medulla is the cause of the epileptic convulsion, it is probably the effect—when it occurs in the inebriate—which the alcohol exerts in producing a transitory spasm of the muscular fibers of the arteries with consequent arterial anæmia.

*Prognosis.* In epilepsy occurring in the inebriate himself, as the result of the morbid irritability produced in the central nervous system by his excessive drunkenness, we may reasonably expect an ultimate cure, if there is no structural change in the brain which has resulted from the course of inebriety. I have had three such cases of recovery of epileptic patients, in the cases under my charge, during the past year. On the other hand, when disease occurs in the offspring of intemperate ancestors, as the result of the hereditary tendency, it depends more certainly upon structural disease of the brain, and, as a general rule, I have found that the more frequent the recurrence of the epileptic convulsions, in such patients, and the deeper the impression which they leave behind them, the less hope is there of ultimate recovery. —*Quarterly Journal of Inebriety.*

